# Genomic insights into head and neck cancer

**DOI:** 10.1186/s41199-016-0003-z

**Published:** 2016-06-03

**Authors:** Tim N. Beck, Erica A. Golemis

**Affiliations:** 1 Program in Molecular Therapeutics, Fox Chase Cancer Center, 333 Cottman Ave, Philadelphia, PA 19111 USA; 2 Program in Molecular and Cell Biology and Genetics, Drexel University College of Medicine, Philadelphia, PA 19129 USA

**Keywords:** Head and neck cancer, TCGA, HPV, Genomics, Cancer therapy, Cell cycle, Personalized medicine, Tumor heterogeneity

## Abstract

Head and neck squamous cell carcinoma (HNSCC) is the sixth most common cancer worldwide and is frequently impervious to curative treatment efforts. Similar to other cancers associated with prolonged exposure to carcinogens, HNSCCs often have a high burden of mutations, contributing to substantial inter- and intra-tumor heterogeneity. The heterogeneity of this malignancy is further increased by the rising rate of human papillomavirus (HPV)-associated (HPV+) HNSCC, which defines an etiological subtype significantly different from the more common tobacco and alcohol associated HPV-negative (HPV-) HNSCC. Since 2011, application of large scale genome sequencing projects by The Cancer Genome Atlas (TCGA) network and other groups have established extensive datasets to characterize HPV- and HPV+ HNSCC, providing a foundation for advanced molecular diagnoses, identification of potential biomarkers, and therapeutic insights. Some genomic lesions are now appreciated as widely dispersed. For example, HPV- HNSCC characteristically inactivates the cell cycle suppressors TP53 (p53) and CDKN2A (p16), and often amplifies CCND1 (cyclin D), which phosphorylates RB1 to promote cell cycle progression from G1 to S. By contrast, HPV+ HNSCC expresses viral oncogenes E6 and E7, which inhibit TP53 and RB1, and activates the cell cycle regulator E2F1. Frequent activating mutations in PIK3CA and inactivating mutations in NOTCH1 are seen in both subtypes of HNSCC, emphasizing the importance of these pathways. Studies of large patient cohorts have also begun to identify less common genetic alterations, predominantly found in HPV- tumors, which suggest new mechanisms relevant to disease pathogenesis. Targets of these alterations including AJUBA and FAT1, both involved in the regulation of NOTCH/CTNNB1 signaling. Genes involved in oxidative stress, particularly CUL3, KEAP1 and NFE2L2, strongly associated with smoking, have also been identified, and are less well understood mechanistically. Application of sophisticated data-mining approaches, integrating genomic information with profiles of tumor methylation and gene expression, have helped to further yield insights, and in some cases suggest additional approaches to stratify patients for clinical treatment. We here discuss some recent insights built on TCGA and other genomic foundations.

## Background

Head and neck squamous cell carcinoma (HNSCC) is the sixth most common cancer, with annual incidence of 600,000 cases worldwide [1]. Anatomically, head and neck cancer regions include the oral cavity, the pharynx (nasopharynx—behind the nose; oropharynx—soft palate, base of the tongue and the tonsils; hypopharynx—the lowest part of the pharynx), the larynx, the paranasal sinuses, the nasal cavity and the salivary glands [2]. Beyond distinction by anatomic sites, HNSCC is divided into two broad classes: human papillomavirus (HPV)-associated (HPV+) and HPV-negative (HPV-) disease. The majority of HPV-negative HNSCC arises from the larynx and oral cavity [3, 4], although a small fraction of cases originates in the oro- and hypopharynx. HPV+ disease is typically found in the oropharynx, with a minority of cases detected in the larynx and oral cavity [5]. As of 2016, the majority of HNSCC is HPV- disease, and is most commonly associated with tobacco use and heavy alcohol consumption [6]. The exception is oropharyngeal HNSCC, 60-70 % of which is HPV+ in North America and Europe (significant geographic variation exists in the prevalence of HPV+ disease worldwide [3, 5, 7]). Over 150 types of HPV have been identified, with HPV subtype 16 (HPV–16) identified as the most oncogenic, detected in over 90 % of HPV+ oropharyngeal cancers [8]. HPV+ HNSCC is typically diagnosed in a younger patient population (6th decade of life; [5, 9]) and its prevalence has dramatically increased since the 1980’s (then only detected in 16 % of oropharyngeal cancer; [7, 9]). HPV- HNSCC is generally diagnosed in an older patient population (7^th^ decade of life), often presents with locally advanced or metastatic features, and has a relatively poor prognosis compared to HPV+ tumors [5, 10].

Both HPV+ and HPV- HNSCC are treated with a combination of surgery, radiation and adjuvant chemotherapy. Treatment specifics vary depending on anatomic site and disease stage. In general, low stage tumors are treated with surgery, followed by radiation if positive surgery margins are detected. For more advanced cases treatment includes surgery, if possible, followed by radiation with or without adjuvant chemotherapy [1, 9, 11]. In spite of significant improvements, including the introduction of targeted and immunotherapies (most prominently, immune checkpoint inhibitors targeting cytotoxic T-lymphocyte-associated antigen 4 (CTLA-4) and programmed cell death protein 1 (PD-1) [12]), as of 2015 the relative 5-year survival rate is only approximately 25–40 % for HPV- and 70–80 % for HPV+ HNSCC [1, 13, 14]. To fully capture the diversity of HNSCC and to gain clinically meaningful insights that can improve treatment, it seems critical to define the full spectrum of molecular alterations and the heterogeneity associated with this pathology.

At no prior point in time has it been possible to describe the molecular landscape of the various, mostly anatomically defined cancers with as much detail and precision as is possible today [15–17], based on concerted efforts to uncover the genomic (most advanced), epigenomic, proteomic and transcriptomic changes that occur as healthy tissue turns malignant, metastatic and resistant to treatment [18]. The Cancer Genome Atlas (TCGA) network and others have periodically published datasets on many cancers [15, 17], including extensive analyses of HPV- and to a lesser degree HPV+ HNSCC (Table 1; [17, 19–24]). Amongst non-lung and non-skin tumor types, head and neck cancer has one of the highest rates of non-synonymous mutations and a high degree of genomic instability [15, 16, 25, 26], which contribute to the enormous heterogeneity of HNSCC [19, 24]. Since large-scale datasets began to appear in 2011 [20, 22], a number of groups have performed integrated bioinformatics, translational, and clinical analyses that leverage the genomic resources, suggesting new research directions. This review summarizes and highlights potential therapeutic opportunities in HPV- and HPV+ HNSCC based on the analysis of high throughput data published by the TCGA network and others.

**Table 1:** High-throughput genomic studies of HNSCC. The most frequently altered genes described in seven studies are shown, separated by HPV status when possible

TCGA (2015; [19])^a^	Seiwert et al. (2015; [21])	Lin et al. (2014; [37])^a^	Pickering et al. (2014; [36])^b^	Pickering et al. (2013; [35])	Stransky et al. (2011; [22])^a^	Agrawal et al. (2011; [20])^a^
HPV-	HPV-	N/A (NPC)	N/A (Tongue)	N/A (OSCC)	HPV-	N/A
*n* = 243	*n* = 69	*n* = 128	*n* = 34 (YT 16, OT 28)	*n* = 35-40	*n* = 63	*n* = 28
TP53 (84 %, M)	TP53 (81 %, M)	TP53 (17 %, M/D)	TP53 (94 %, 57 %, M)	CDKN2A (74 %, D)	TP53 (73 %, M)	TP53 (79 %, M)
CDKN2A (57 %, M/D)	CDKN2A (33, M/D)	CDKN2A/B (13 %, M/D)	CSMD1 (25 %, 75 %, D)	TP53 (66 %, M)	CDKN2A (25 %, M/D^c^)	NOTCH1 (14 %, M)
let-7c (40 %, miRNA)	MDM2 (16 %, A)	ARID1A (11 %, M/D)	PIK3CA (0 %, 11 %, M); (30 %, 70 %, A)	FAT1 (46 %, M/D)	SYNE1 (22 %, M)	RELN (14 %, M)
PIK3CA (34 %, M/A)	MLL2 (16 %, M)	SYNE1 (8 %, M)	CDKN2A (6 %, 4 %, M); (55 %, 65 %, D)	TP63 (26 %, A)	CCND1 (22 %, A^c^)	SYNE1 (14 %, M)
FADD (32 %, A)	NOTCH 1 (16 %, M)	ATG13 (6 %, M/D)	FADD/CCND1 (40 %, 65 %, A)	CCND1 (23 %, A)	MUC16 (19 %, M)	EPHA7 (11 %, M)
FAT1 (32 %, M/D)	CCND1 (13 %, A)	MLL2 (6 %, M)	FAT1 (6 %, 25 %, M); (50 %, 35 %, D)	MAML1 (23 %, D)	USH2A (18 %, M)	FLG (11 %, M)
CCND1 (31 %, A)	PIK3CA (13 %, M)	PIK3CA (6 %, M/A)	EGFR (20 %, 50 %, A)	EGFR (17 %, A)	FAT1 (14 %, M)	HRAS (11 %, M)
NOTCH1/2/3 (29 %, M/D)	PIK3CB (13 %, M/A)	CCND1 (4 %, A)	NOTCH1 (25 %, 18 %, M)	TNK2 (17 %, A)	LRP1B (14 %, M)	PIK3AP1 (11 %, M)
TP63 (19 %, A)	UBR5 (13 %, M/D)	NOTCH3 (4 %, M)	HLA-A (0 %, 14 %, M)	AKT1 (14 %, A)	ZFHX4 (14 %, M)	RIMBP2 (11 %, M)
EGFR (15 %, M/A)	EGFR (12 %, A)	FGFR2 (4 %, M)	CASP8 (6 %, 11 %, M)	SRC (14 %, A)	NOTCH1 (13 %, M)	SI (11 %, M)
HPV+	HPV+				HPV+	HPV+
*n* = 36	*n* = 51				*n* = 11	*n* = 4
E6/7 (100 %)	E6/7 (100 %)				E6/E7 (100 %)	E6/E7 (100 %)
PIK3CA (56 %, M/A)	PIK3CA (22 %, M)				PIK3CA (27 %, M)	EPHB3 (25 %, M)
TP63 (28 %, A)	TP63 (16 %, M/A)				RUFY1 (18 %, M)	UNC5D (25 %, M)
TRAF3 (22 %, M/D)	PIK3CB (13 %, M/A)				EZH2 (18 %, M)	NLRP12 (25 %, M)
E2F1 (19 %, A)	FGFR3 (14 %, M)				CDH10 (18 %, M)	PIK3CA (25 %, M)
let-7c (17 %, miRNA)	NF1/2 (12 %, M)				THSD7A (18 %, M)	TM7SF3 (25 %, M)
NOTCH1/3 (17 %, M)	SOX2 (12 %, A)				FAT4 (18 %, M)	ENPP1 (25 %, M)
FGFR3 (11 %, F/M)	ATM (10 %, D)				KMT2D (18 %, M)	NRXN3 (25 %, M)
HLA-A/B (11 %, M/D)	FLG (12 %, M)				ZNF676 (18 %, M)	MICAL2 (25 %, M)
EGFR (6 %, M)	MLL3 (10 %, M)				MUC16 (18 %, M)	

*N/A* HPV status not available, *NPC* nasopharyngeal cancer, *YT* young tongue, *OT* old tongue, *OSCC* oral squamous cell carcinoma, *M* mutation, *A* amplification, *D* deletion, *F* fusion

^a^Data was accessed using cBioportal [38, 39]

^b^values for A and D are approximations

^c^percentages are not based on the 63 cases, because CNAs were not analyzed for all cases

### Foundational genomic datasets

The pathophysiological differences between HPV+ and HPV- HNSCC necessitate that genomic analyses apply rigorous classification methods for HPV dependence in clinical samples [10, 22, 27]. HPV status is most commonly determined by polymerase chain reaction (PCR) or *in situ* hybridization (*ISH)* to detect HPV genetic material, or by immunohistochemical (IHC) staining for the tumor suppressor p16 (CDKN2A), which is induced as a consequence of HPV-associated transformation [28]. p16 IHC staining is greatly increased as a result of HPV infection, and is a reliable proxy for positive HPV status in primary tumors of the oropharynx [28]. Virally encoded proteins target the cell cycle regulator retinoblastoma 1 gene (RB1), providing one potential feedback mechanisms for enhancing expression of p16 ([29], and discussed further below). Alternatively, it has been shown that upregulation of p16 can also occur as a cellular response to the infection itself, through induction of the histone 3 lysine-27 (H3K27) specific demethylases KDM6A and KDM6B [30–32]. For other anatomic sites, the true positive rate for p16 IHC staining falls below 50 %, reflecting the rarity of HPV-associated tumors outside of the oropharynx [28, 33]. High p16 expression also occurs in about 5 % of HPV- cases, for reasons that are at present unclear [28]. For these reasons, the TCGA network took extensive measures to ensure proper HPV classification of each tumor: in addition to p16 staining and *ISH*, whole HPV genome sequencing as well as HPV RNA-Seq was performed. HPV positive cases were classified as such if > 1000 RNA-Seq reads aligned to viral genes E6 and E7 [19].

The TCGA network analyzed 243 HPV-negative and 36 HPV-positive tumors using multiple platforms (RNA sequencing, DNA sequencing, reverse phase protein array (RPPA), DNA methylation profiling and miRNA sequencing) to define the molecular landscape of this malignancy [19, 34]. Most of the patients in the TCGA cohort were male (~70 %) and heavy smokers (51 mean pack years; [19]), closely resembling the general HPV- HNSCC patient population [1, 11]. Tumors predominantly originated from the oral cavity (*n* = 172; 62 %; 160/172 HPV- and 12/172 HPV+) and the larynx (*n* = 72; 26 %; 71/72 HPV- and 1/72 HPV+), with only a few cases originating from the oropharynx (*n* = 33; 12 %; 11/33 HPV- and 22/33 HPV+) and only two from the hypopharynx (1/2 HPV+ and 1/2 HPV-).

Beyond the work of the TCGA network, additional genomic sequencing studies (Table 1) were performed by Stransky et al. (53 HPV- and 11 HPV+; [22]), Agrawal et al. (28 HPV- and 4 HPV+; [20]), Pickering et al. (40 oral squamous cell carcinoma; likely HPV-negative; [35]), Seiwert et al. (69 HPV- and 51 HPV+; [21]), and Pickering et al. (34 squamous cell carcinoma of the oral tongue; likely HPV-negative; [36]). These predominantly relied on a single platform (exome/massively parallel sequencing) for data acquisition. In addition, Lin et al. have sequenced 128 cases of nasopharyngeal carcinomas (NPC; likely HPV-; [37]).

The TCGA dataset [19] is conveniently accessible through cBioportal [38, 39], as are the datasets from Stransky et al., Agrawal et al. [20, 22], and the NPC study [37]. The 279 patient TCGA dataset provides the most extensive tumor profiles, including mutational data from whole exome sequencing, identification of somatic copy number alterations using the GISTIC algorithm [40], mRNA expression data (RNA-Seq V2 RSEM), and protein expression data for a total of 165 combined phosophoproteins and proteins (reverse-phase protein array/microarray; [38, 39, 41]). The other three datasets accessible through cBioPortal predominantly cover somatic mutations. The dataset for Pickering et al. [35] is available through Gene Expression Omnibus (GEO; [42]); the remaining studies provide access to datasets via links provided in the original publications. Table 1 summarizes the top alterations detected in each study for HPV- and, if available, HPV+ cases. Certain alterations were detected across several studies; whereas, a significant number of alterations were not uniformly detected, potentially due to the variation in detection platforms, disease heterogeneity and significant demographic differences.

### Common genomic defects: HPV+ and HPV-

Copy number alterations (CNA) are frequent in HNSCC [26] and are highly concordant across most of the genome for HPV- and HPV+ cases [19, 43]. One of the most frequently amplified regions (in approximately 15–30 % of cases [19]) is on chromosome 3q and includes the anti-apoptotic kinase protein kinase C (PIK3CA), and the transcription factors TP63 and SOX2 [44]. Additional amplifications found in both the HPV+ and HPV- disease subtypes include chromosomes 5p and 8q [19, 43], which encompass telomerase TERT (5p) and the oncogene MYC (8q). Commonly seen deletions prominently cover parts of chromosome 3p and 8p, impacting two tumor suppressor genes: FHIT (3p; expression loss is associated with worse survival in HNSCC [45]) and CSMD1 (8p; [19, 46, 47]). Losses in 3p and 8p and gains in 3q, 5p and 8q are also frequently seen in squamous cell carcinomas (SQCC) of the lung [48], highlighting important genomic similarities between SQCC and HNSCC [19, 43, 48].

**Fig. 1:**
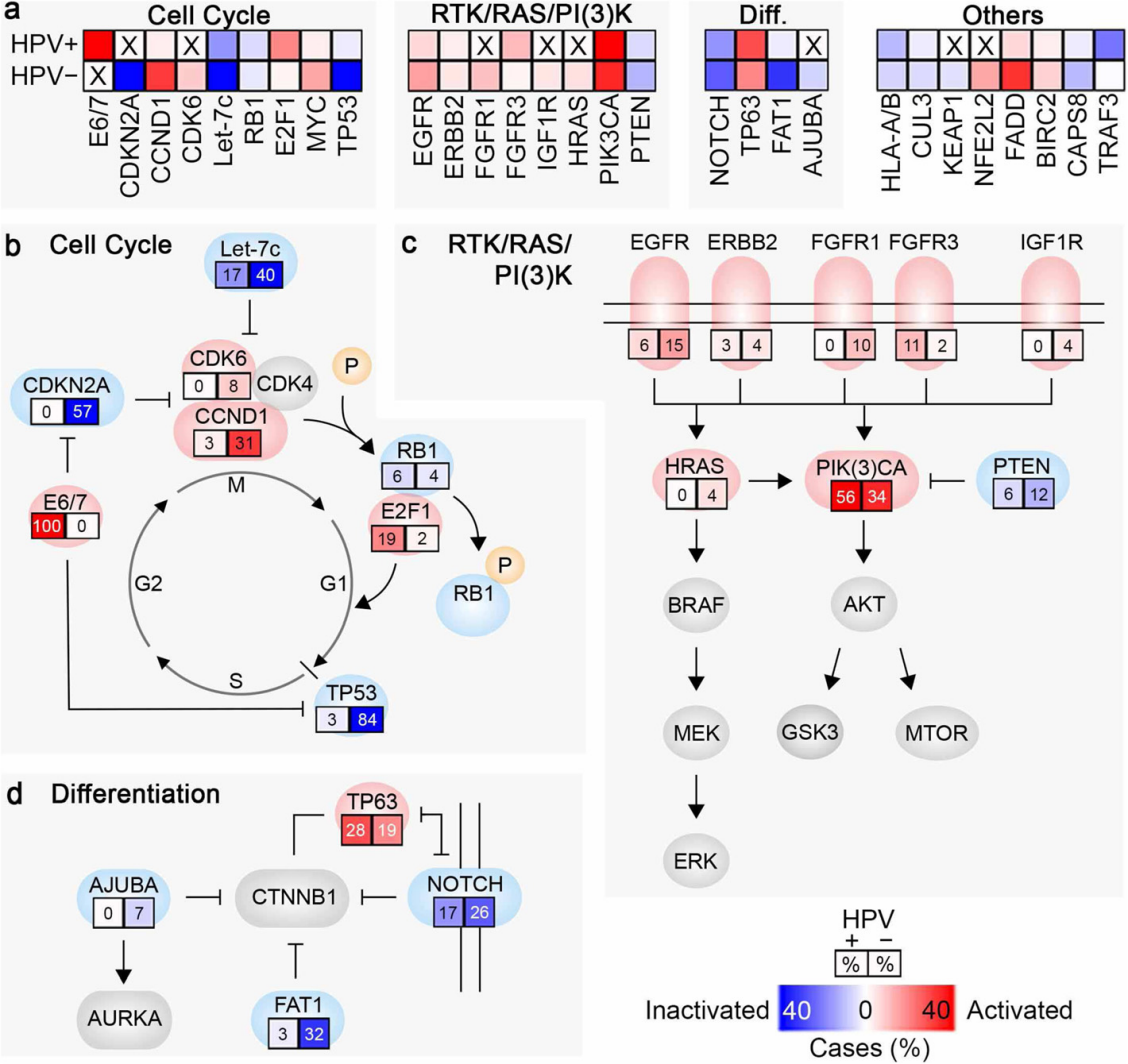
The genomic profile of HNSCC. **a** Percent alteration for each listed gene in HPV+ and HPV- tumors (x = 0 % of cases); dominant alterations in **b** cell cycle, **c** RTK/RAS/PI(3)K signaling, and **d** differentiation associated genes. Percentages are based on the The Cancer Genome Atlas Network dataset [19]. Diff. = differentiation; RTK = receptor tyrosine kinase

The microRNA let-7c, a cell cycle regulator, is frequently inactivated in both HPV- and HPV+ HNSCC in the TCGA cohort (Fig. 1a and b). Depressed expression of let7-c is associated with increased expression of CDK4, CDK6, E2F1 and PLK1, kinases and translational regulators important for progression through the cell cycle [19, 49]. In depth analysis of TCGA microRNA data has been used to test the hypothesis that expression of 28 microRNAs selected based on *in vitro* experiments could predict response to radiotherapy [50]. Patients from the TCGA cohort with complete clinical annotations were divided into three groups: radiation with complete response (radiosensitive), radiation with tumor progression (radioresistant), and not irradiated. This analysis suggested that upregulation of miR-016, miR-29, miR-150, miR-1254 and downregulation of let-7e correlated with complete response to radiotherapy. Effects were linked to ATM expression. Higher levels of ATM correlated with increased radio-resistance, based on RPPA data also provided by the TCGA [50]. These interesting findings necessitate validation using additional cohorts, but clearly indicate the potential value of analyzing microRNA expression in HNSCC.

One of the most commonly activated (mutationally or due to amplification of the 3q chromosomal region) genes in both HPV+ and HPV- cases in the TCGA cohorts (56 and 34 %, respectively) and other studies (Table 1; [21–23]) is PIK3CA, encoding the p110α catalytic subunit of phosphinositol-3-kinase (Fig. 1; [19]). In this regard, HNSCC is similar to many other cancers in which PIK3CA is amongst the most commonly mutated genes [18]. PIK3CA encodes a lipid kinase that regulates signal propagation from multiple input sources [51], including many of the receptor tyrosine kinases (RTKs) relevant to HNSCC (Fig. 1c; [19, 52, 53]). Functionally, PIK3CA regulates phosphorylation of AKT1, and mutated PIK3CA has been shown to attenuate apoptotic signals and support tumor invasion [54]. Additionally, mutationally activated PIK3CA has been shown to support cyclin D activity [55]; thus, further emphasizing the tremendous relevance of cell cycle dysregulation in head and neck cancers [43, 56].

Lui et al. performed a focused whole-exome analysis of 151 HNSCC tumors (datasets from Stransky et al. and Agrawal et al., plus 45 additional cases [20, 22]), specifically exploring PI3K pathway mutations for therapeutic opportunities [23], This analysis (which did not assess CNAs) indicated the PI3K pathway was the most frequently mutated oncogenic pathway (30.5 % of tumors, 46/151 [23]). PIK3CA in particular was mutated in 12.6 % of cases (19/151; [23]), which is substantially less than the number of cases with mutated PIK3CA (21 %, 58/279) reported by the TCGA [19]. Nevertheless, assessment of patient derived xenografts expressing wild type PI3KCA or mutant PI3KCA and treated with vehicle or the mTOR/PI3K inhibitor BEZ-235 [57] indicated tumors with mutated PI3KCA were exquisitely sensitive to the small molecule inhibitor, whereas tumors without the mutation did not respond to the treatment [23]. Several studies using pre-clinical models also demonstrated that HNSCC with wild type PI3KCA is sensitive to PI3K/mTOR inhibitors, particularly in combination with a MEK inhibitor or in combination with radiation in the context of wild type p53 [58, 59]. Development of a large number of PI(3)K inhibitors is ongoing, with several promising compounds currently being tested in clinical trials [60].

Another commonly altered gene in HNSCC is NOTCH1. NOTCH1 is a transmembrane kinase frequently mutationally inactivated (most commonly via missense or truncating mutation) in both HPV+ and HPV- cases (13–26 % and 8–17 %, respectively [19, 21]; Table 1). The role of the NOTCH pathway is complicated and depends on the overall organization of the broader signaling network and on the specific tissue type [61]. Exome sequencing of HNSCC strongly implicated NOTCH1 as a tumor suppressor in this malignancy, as close to 40 % (11/28) of NOTCH1 mutations were truncating mutations predicted to be inactivating [20]. This conclusion was supported by the observation that NOTCH1 knockout mice developed tumors due to increased oncogenic CTNNB1 signaling [62]. Additional work in tongue carcinoma cells observed robust down-regulation of CTNNB1 in the background of stable expression of NOTCH1 [63]. Another important feature of NOTCH1 is its participation in reciprocal negative regulation with p63 [64], a member of the p53 family found to be activated with high frequency in HNSCC (19 and 28 % in HPV- and HPV+ cases respectively, mostly due to amplification [19]). In keratinocytes, overexpression of p63 induced cell growth in part by suppression of p21 and thus directly counteracting the growth suppressive input from NOTCH1 [65]. The TCGA data supports this NOTCH1-p63 paradigm in HNSCC, given the high incident of NOTCH1 inactivating mutations and the significant incident of p63 activation. Of note, p63 is transcriptionally activated by two distinct promoters [66]; one of the two resulting p63 variants contains an N-terminal transactivation domain (TAp63), whereas, the other transcript lacks the N-terminal domain and is termed ΔNp63 [66]. The two p63 isoforms are functionally distinct [67], with ΔNp63 acting as a dominant negative regulator of p53 and with TAp63 opposing cell cycle arrest and apoptosis [66, 68]. ΔNp63 is highly expressed in HNSCC [69] and indeed inhibits NOTCH1 activity [65, 70].

### HPV-Negative HNSCC

Well prior to the advent of high throughput sequencing, alterations in several genes, including inactivation or deletion of the tumor suppressors CDKN2A (p16; [71]) and TP53 (p53; [72]), and overexpression (via amplification and elevated transcription) of the epidermal growth factor receptor EGFR [73] had been identified as relevant to the pathogenesis of HPV-negative HNSCC. Based on TCGA analysis of genomic-scale data, the two most commonly inactivated genes in HPV- tumors were confirmed as TP53 (84 % of cases [19]; a percentage similar to the one reported by Seiwert et al. (81 % of 69 HPV- tumors; [21]) and Stransky et al. (73 % of 63 HPV- tumors; [22])) and CDKN2A (4–74 % of cases Fig. 1a and b and Table 1; [19, 21, 22]; the broad range is in part due to the lack of CNA data for several of the studies and the difficulties associated with sequencing of GC-rich regions, which are found in CDKN2A [74, 75]).

Due to the high frequency of mutations in TP53 (Table 1), significant effort has been focused on elucidating the prognostic potential of this gene. Some work suggests improved overall survival for patients with wild type TP53 compared to patients with TP53 mutations predicted to be functionally disruptive (i.e., nonsense mutations or missense mutations disruptive to the L2 or L3 DNA-binding domains [76]). Other reports indicated that TP53 status is of low prognostic value when considered independently from other variables [77]. A multi-tiered genomic analysis of 250 HPV-negative tumors in 2014 (TCGA dataset; approximately corresponding to the cohort described above) confirmed that disruptive TP53 mutations correlated with reduced survival; however, in this analysis, cases with TP53 mutations predicted to be non-disruptive also had significantly worse survival outcomes compared to cases with wild type TP53 [78]. Strikingly, this study identified TP53 mutations as frequently co-occurring with deletions of chromosomal region 3p (179 out of 250 cases), with the combination associated with significantly worse survival than was predicted for TP53 mutations or 3p deletions considered independently [78]. Further stratification of the 179 TP53-3p cases showed that elevated expression of miR-548k (a microRNA encoded by a gene proximal to cyclin D1 (CCND1) and the death receptor FADD at 11q13 and described as oncogenic in esophageal squamous cell cancer [79]) predicted further reduction in survival [78].

Efforts to elucidate the prognostic value of different TP53 mutations have also led to the development of a novel computation approach termed the Evolutionary Action score of TP53-coding variants (EAp53; [80–82]). EAp53 stratifies HNSCC patients with tumors harboring TP53 missense mutations based on an estimated degree of risk assigned to each mutation. The foundational principles of this approach are based on previously identified TP53 “gain of function” mutations that enhanced cell transformation and chemotherapy resistance [83]. EAp53 assigns functional sensitivity to sequence variations based on evolutionary substitutions for every sequence position and calculates if substitutions correlate with larger or smaller phylogenetic divergences to determine “risk” [80, 82, 84]. HNSCC patients with p53 mutations classified using EAp53 as high-risk had significantly worse survival outcomes and reduced periods until distant metastases developed [80], as well as increased resistance to chemotherapy [81]. As larger datasets with clinical annotations become available, it will be critical to refine and validate these models, and to determine if and how TP53 status is suitable to predict efficacy of different therapeutic interventions.

CDKN2A regulates cell cycle progression by blocking the activity of CCND1 (cyclin D1) and its associated kinases, CDK6 and CDK4, which phosphorylate and inactivate the tumor suppressor RB1 (Fig. 1b; [56, 85, 86]). Inactivation of the CDKN2A gene was found in 57 % of HPV- cases in the TCGA cohort [19]; however, other studies produced discordant values for genomic alterations of CDKN2A (ranging from 4 to 74 %; [20–22, 35, 36]; Table 1). Evaluation of CDKN2A status is somewhat complicated by the fact that the gene is GC-rich (> 60 % of bases are cytosine or guanine; [87]). Sequencing GC-rich regions can be problematic because of their higher melting temperature compared to GC-low regions, which is due to base stacking and more stable secondary structure [74, 75]. Methylation-associated inactivation of CDKN2A (further discussed below) is another important factor potentially complicating assessment of the function status of this gene [88–91]. Direct comparison of cases in the TCGA cohort with homozygous deletions or predicted inactivating mutations in CDKN2A versus wild type CDKN2A did not indicate a survival difference. However, as with TP53, subsequent refined analysis of CDKN2A status emphasized that the patient cohort with low mRNA expression of CDKN2A (RNA-seq: z < 3-fold) did have reduced survival (*p* = 0.037; [56]). This observation is in accordance with other work that indicated improved survival for patients with p16-positive non-oropharyngeal squamous cell carcinoma [92]. Further emphasizing the importance of this signaling axis is the fact that CCND1 is the most frequently amplified gene in the TCGA cohort of HPV- HNSCC cases, detected in 31 % of cases (confirming earlier studies [19, 21, 22, 56]). Beck et al. reported that high RNA expression of CCND1 (z > 2-fold) not only correlated with reduced survival in the TCGA dataset [19, 56], but also co-occurred frequently with CDKN2A deletions (co-occurrence ratio: 0.817). Cases harboring both, amplified CCND1 and deleted CDK2N2A had much worse prognosis than cases without these alterations [56].

In the TCGA cohort, EGFR was amplified in 12 % of HPV- cases [19], the same % of cases with EGFR amplification was reported by Seiwert et al. [21]. Seiwert et al. did not report significant incidence of alteration in HER2 (also known as ERBB2), ERBB3 and ERBB4 and the TCGA also only detected alterations of those genes in a small number of cases (4–6 %; [19]). Nevertheless, alterations in ERBB2 or ERBB3 have been directly linked to resistance to EGFR-targeted therapy and are thus of therapeutic relevance [93]. Mining of TCGA data highlighted that RPPA expression of pHER2 correlated with expression of HER2, and both, pHER2 and HER2 expression correlated with protein expression of EGFR [93], providing some patient data in support of *in vivo* results in which dual kinase inhibition of EGFR and HER2 enhances response to cetuximab [94]. In addition, the RTKs FGFR1 and IGF1R were identified with activating mutations in 10 and 4 % of HPV- HNSCC, respectively, while no mutations of these kinases were identified in HPV+ HNSCC tumors (Fig. 1a and c). FGFR1 and IGFR1 participate in a signaling network that includes EGFR and other ERBB family members (Fig. 1c), and both can contribute to resistance to EGFR-targeted therapeutics, the only type of targeted therapy approved for HNSCC [73, 95, 96]. Functioning downstream of these RTKs, the GTPase HRAS was almost exclusively altered in HPV- HNSCC (5 %), propagates pro-proliferation and pro-survival signaling via the BRAF-MEK-ERK axis, and provides alternative input to activate PI3K (Fig. 1c; [97–99]).

For two additional genes, AJUBA and FAT1, almost all detected alterations were found in HPV- tumors (Fig. 1a and d). Both genes are involved in differentiation and are linked to the NOTCH/CTNNB1 signaling pathway as negative regulators [19, 100, 101]. The scaffolding protein AJUBA, inactivated in 7 % of HPV- cases (0 % of HPV+ tumors), has also been implicated in interactions with Aurora-A kinase (AURKA), a critical regulator of mitosis [102]. AURKA is overexpressed in a significant percentage (7 %) of HNSCC cases and correlated with diminished survival in an analysis of provisional TCGA data (significant overlap with the published TCGA dataset; [19, 103]). FAT1 is a member of the cadherin-like protein family and has been described as a suppressor of cancer cell growth based on a role in binding to and antagonizing CTNNB1 [100]. FAT1 had previously been shown to be mutated in roughly 7 % of 60 head and neck tumors [100], but was detected to be inactivated (missense/truncating mutations and homozygous deletions) in a much greater percentage of HPV- cases (32 %; versus inactivated in only 3 % of HPV+ cases; Fig. 1a and d) analyzed by the TCGA network [19]. The discrepancy may be due to a number of reasons, including differences in sample processing, determination of HPV status, demographic factors, different acquisition platforms, and differently constructed analytical pipelines.

The TCGA analysis also identified a set of less well studied alterations associated with oxidative stress, specifically involving CUL3, KEAP1 and NFE2L2 [19]. KEAP1 and NFE2L2 were exclusively altered in HPV- HNSCC (Fig. 1a). KEAP1 was inactivated in 5 % of cases and NFE2L2 was activated in 14 % of cases. Functionally, NFE2L2 is a transcription factor that regulates antioxidant and stress-responsive genes [104]. KEAP1 complexes with the E3 ligase CUL3 (inactivated in 6 % of HPV- cases) to polyubiquitinate NFE2L2 [105]; thus, disruption of canonical KEAP1-CUL3 function promotes NFE2L2 activity [19]. Intriguingly, in lung cancer, a NFE2L2-centric gene signature has been proposed as a valuable prognostic biomarker [106]. This may be relevant because significant molecular similarities between HNSCC and lung squamous cell cancers (SQCC) exist [19, 43, 48, 107], including shared dysregulation of KEAP1 and NFE2L2. Secondary analysis of the TCGA dataset indeed revealed that DNA level alterations of any member of the KEAP1/CUL3/RBX1 complex correlated with significantly reduced survival (median survival of ~35 months versus ~72 months; [108]).

Lastly, the TCGA network detected co-amplification of chromosome regions 11q13 and 11q22. Found within region 11q22, an amplicon previously described in lung, esophageal and cervical cancer [109–111], are the coding sequences for BIRC2 and YAP1. BIRC2 encodes c-IAP1 and is a member of the inhibitor-of-apoptosis family [112]. Functionally, BIRC2 inhibits caspase activity, including the activity of CASP8 [112], and it has been shown that BIRC2 plays an important role in the ubiquitination and degradation of TRAF3 (tumor necrotizing factor receptor-associated factor 3), a negative regulator of NF-kB activity [113]. BIRC2 is more commonly altered in HPV- HNSCC (7 % of cases versus 3 % of cases in HPV+ HNSCC) and, as would have been predicated based on functionality, CASP8 was also frequently detected as inactivated through mutations or homozygous deletion (11 % of HPV- cases; Fig. 1a). YAP1 is a proto-oncogenic transcription factor downstream of BIRC2 and associated with the Hippo pathway [114]. Amongst cancers analyzed by the TCGA, HNSCC had the fifth highest incident of amplified YAP1 (6.3 % of cases). Interestingly, a recent study found that YAP1 amplification strongly correlated with resistance to cetuximab in vitro [115], which may reflect YAP1 associated upregulation of the EGFR ligand amphiregulin; further investigations are needed to fully uncover the precise mechanism of this type of resistance [115, 116]. Amplification of region 11q13 includes the region encoding the Fas-associated death domain gene (FADD; established as frequently overexpressed in HNSCC [117] and found to be amplified in 32 % of HPV- cases analyzed by the TCGA [19]). Importantly, FADD has been implicated in increased lymph node metastasis in HNSCC [117].

### HPV-Positive HNSCC

At the molecular level, HPV+ carcinomas significantly differ from HPV- cases, highlighted in great detail by the TCGA network and others [10, 19, 21, 27, 92]. A significant limitation of the TCGA study is the fact that only 36 HPV+ cases were analyzed [19], a limitation partially compensated for by the work of other groups (Table 1; [21, 23, 34, 118]). Further analysis of additional tumors is clearly needed; however, some conclusions can be made in spite of the limited numbers of cases.

HPV+ HNSCC is defined by infection of tumor cells with HPV. HPV DNA can exist either integrated into the human genome or in a nonintegrated form [34, 119]. Upon infection, the HPV genome (8 kb) is first amplified as extrachromosomal circular elements (episomes), some of which may subsequently integrate into one or more location within the host genome [119]. It has been reported that HPV integration sites are randomly distributed throughout the genome [120]. In one study, analysis of 35 of the 36 HPV+ TCGA HNSCC cases identified HPV integration in 25 cases and uncovered distinct gene expression and methylation patterns for HPV integrated versus non-integrated HNSCC, suggesting different pathogenic mechanisms [34]. Another study published similar results for essentially the same group of patients (36 HPV+ HNSCC), and detected HPV DNA integration in 24/36 cases [119]. The general observation regarding HPV integration is not unique to HNSCC, as HPV+ cervical cancers include HPV integrated and non-integrated cases [121]. Compared to episomal HPV DNA, transcripts derived from integrated viral DNA have been shown to be more stable and more strongly associated with increased proliferative capacity of affected cells [122]. It is likely that HPV integration, particularly if within or proximal to key cancer related genes, is important but not essential for the oncogenicity of HPV: the better understood oncogenic contribution of the virus is the production of different oncoproteins [34].

HPV oncoproteins include E6 and E7 (Fig. 1b; [122]), which perform complementary actions in eliminating negative regulators of the cell cycle. E6 binds p53 and targets this tumor suppressor for proteosomal degradation [123]. Tumor suppressor RB1 interacts with E7, which targets RB1 for degradation through association with the cullin 2-ubiquitin-ligase complex [124–126]. As E6 and E7 function through cell cycle dysregulation by eliminating RB1 and TP53, very few alterations in additional cell cycle regulators occur in HPV+ disease: inactivation of CDKN2A, or TP53, or overexpression of CDK6 or CCND1, occur seldom in HPV+ HNSCC. One exception is the transcription factor E2F1, which is normally inhibited by RB1 (Fig. 1b; [56]); it is the only cell cycle regulator identifier by the TCGA study as being predominantly altered in HPV+ cases (19 % activated via amplification of chromosome 20q11, seen in only in 2 % of HPV- HNSCC; Fig. 1a and b).

Also associated with HPV+ disease is the RTK FGFR3, which is activated in 11 % of cases through either mutation or a gene fusion event, and the aforementioned TNF receptor associated factor TRAF3, inactivated in 22 % of cases (versus 1 % in HPV- disease). The FGFR3 fusion partner is TACC3, a protein critical for nucleation of microtubules at the centrosome [127], aberrantly expressed in some cancers and potentially targetable with small molecules [128]. A FGFR3-TACC3 fusion was first described in glioblastoma [129] and subsequently detected in nasopharyngeal carcinoma and other HNSCCs [130, 131]. This fusion event was detected in two of the 36 HPV+ and zero of the HPV- HNSCC TCGA cases [19]. Constitutive kinase activity of the FGFR3-TACC3 oncogene induces loss of mitotic fidelity and leads to aneuploidy [129, 131]. In cases where present, FGFR3-TACC3 appears to be tumor driving and patients are likely to disproportionally benefit from FGFR3 targeting therapy [131]. TRAF3 has mostly been studied in immunological processes and one of its main functions is regulation of NFkB activity [132]. In subsequent studies in HNSCC, functional analysis of TRAF3 has suggested a tumor suppressive role of the gene when overexpressed, and increased cell proliferation in the context of depleted TRAF3 [133].

### Tumor heterogeneity

HPV- and HPV+ HNSCC share one particularly challenging feature: tumor heterogeneity [24]. This aspect of tumor biology has garnered significant attention in recent years because of the immense clinical implications in terms of prognosis, drug resistance and precision medicine [134–136]. Extensive analysis of TCGA data indicates that it is of high relevance in HNSCC [24, 137]. One approach to study HNSCC heterogeneity is based on whole-exome sequencing (WES), which can be used to determine the fraction of total sequenced DNA that contains a given mutant allele: termed mutant-allele fraction (MAF). The width of MAF distribution, normalized to the median MAF value, constitutes the quantitative value of intra-tumor heterogeneity, and has been termed mutant-allele tumor heterogeneity (MATH; [137, 138]). Earlier work indicated that HPV- tumors had significantly higher heterogeneity than HPV+ tumors (though substantial even for HPV+ cases; [137]), which would be predicated based on the frequency of and genomic instability associated with TP53 mutations, increased age and continuous tobacco use [24].

Provocatively, in a ten-variable multivariate analysis of TCGA HNSCC data incorporating MATH scores, no prognostic significance of HPV status, N classification or TP53 mutational status was determined [138]. While the lack of significance in the multivariate analysis does not suggest irrelevance of the three parameters, it strongly suggests that further work is needed to unravel these variables and to determine how much each parameter truly impacts disease progression and survival in the context of appreciated heterogeneity. For example, disruptive TP53 mutations [78] are strongly associated with higher intra-tumor heterogeneity as calculated by MATH (i.e., high MATH scores), and both TP53 mutational status and high MATH scores, based on univariate analysis, indicated reduced survival [78, 137].

Additional innovative and detailed analysis by McGranahan et al. utilized TCGA datasets for nine tumor types, including HNSCC, to highlight important aspects of cancer evolution and clonality [24]. In order to determine if specific alterations were clonal (present in most/all tumor cells sequenced and therefore considered “early” mutations) or subclonal (present in a small fraction of cells and considered “late” mutations) McGranahan et al. used exome sequencing data and single-nucleotide polymorphism arrays to calculate the confidence interval of the cancer cell fraction (CCF; proportion of cancer cells harboring a given mutation; [139–141]) for a given mutation. A 95 % confidence interval of ≥ 1 was used to define clonal (“early”) mutations, and mutations with a confidence interval of less than 1 were defined as subclonal (“late”) mutations [24, 140–142]. In HNSCC, the majority of driver mutations were clonal, and CDKN2A and TP53 were identified as almost exclusively clonal. Based on the proportion of mutations, three mutational signatures (previously defined [25]) were identified for HNSCC: 1) a signature with C > T transitions at CpG sites associated with spontaneous deamination of methylated cytosines that strongly correlated with patient age at diagnosis and was most prevalently linked to “early” mutations; 2) a signature indicative of up-regulation of APOBEC cytosine deaminases [143], seen in both “early” and “late” mutations, although with significant prominence in “late” mutations; and 3) a signature associated with smoking induced mutations, seen predominantly with “early” mutations [24]. McGranahan et al. did not differentiate between HPV+ and HPV- cases, which future studies should do, particularly given that previous analysis of TCGA data detected evidence that HPV infection was strongly associated with APOBEC-mediated mutagenesis in HNSCC [144]. Furthermore, the same study suggested that APOEC-mediated mutagenesis significantly contributes to helical domain E545K and E542K gain of function mutations in PIK3CA, one of the most frequently altered genes in HNSCC (32 out of 58 PIK3CA mutations in the TCGA cohort are E545K/E542K mutations; [19, 21, 144, 145]).

As an emerging concept for this disease, consideration of germline variants may be relevant to fully appreciate tumor heterogeneity. Recent analysis of TCGA data suggested that 15 % (44 out of 291 cases; incompletely congruent with the published TCGA dataset [19]) of HNSCC cases have rare germline truncations, including truncations in several genes important in the Fanconi Anemia Pathway, specifically FANCA and FANCM, which are involved in DNA repair [146, 147]. FANCM mutations significantly correlated with increased somatic mutational frequency in the complete HNSCC cohort (mean age was 60.9 +/− 12.4 years; based on personal correspondence with authors), whereas, FANCA had a similar correlation with the frequency of somatic mutations, but specific in cases defined as younger age (mean age was 46.3 +/− 7.0 years) of onset (no indication regarding HPV status; [146]). Recent studies using murine models have implicated MYH9 as a gene that induces oral squamous cell carcinoma in the context of germline mutations or knockout [148, 149]. MYH9 encodes for non-muscle myosin II-A (NM II-A), best known for its roles as a cytoskeletal protein and during embryonic development [150]. Intriguingly, MYH9 may also acts as a tumor suppressor, by regulating stabilization and nuclear retention of p53 [149]. MYH9 and MYH10 were mutated in 4 and 5 % of cases, respectively within the TCGA cohort of 279 patients [148]. No correlation with HPV status was detected. Success of future clinical efforts, particularly for targeted therapeutics, will likely heavily depend on consideration of heterogeneity and cancer evolution, guided by studies of spatio-temporal differences in genomic alterations, including presence or absence of germline mutations [134, 151].

### Therapeutic insights

**Fig. 2:**
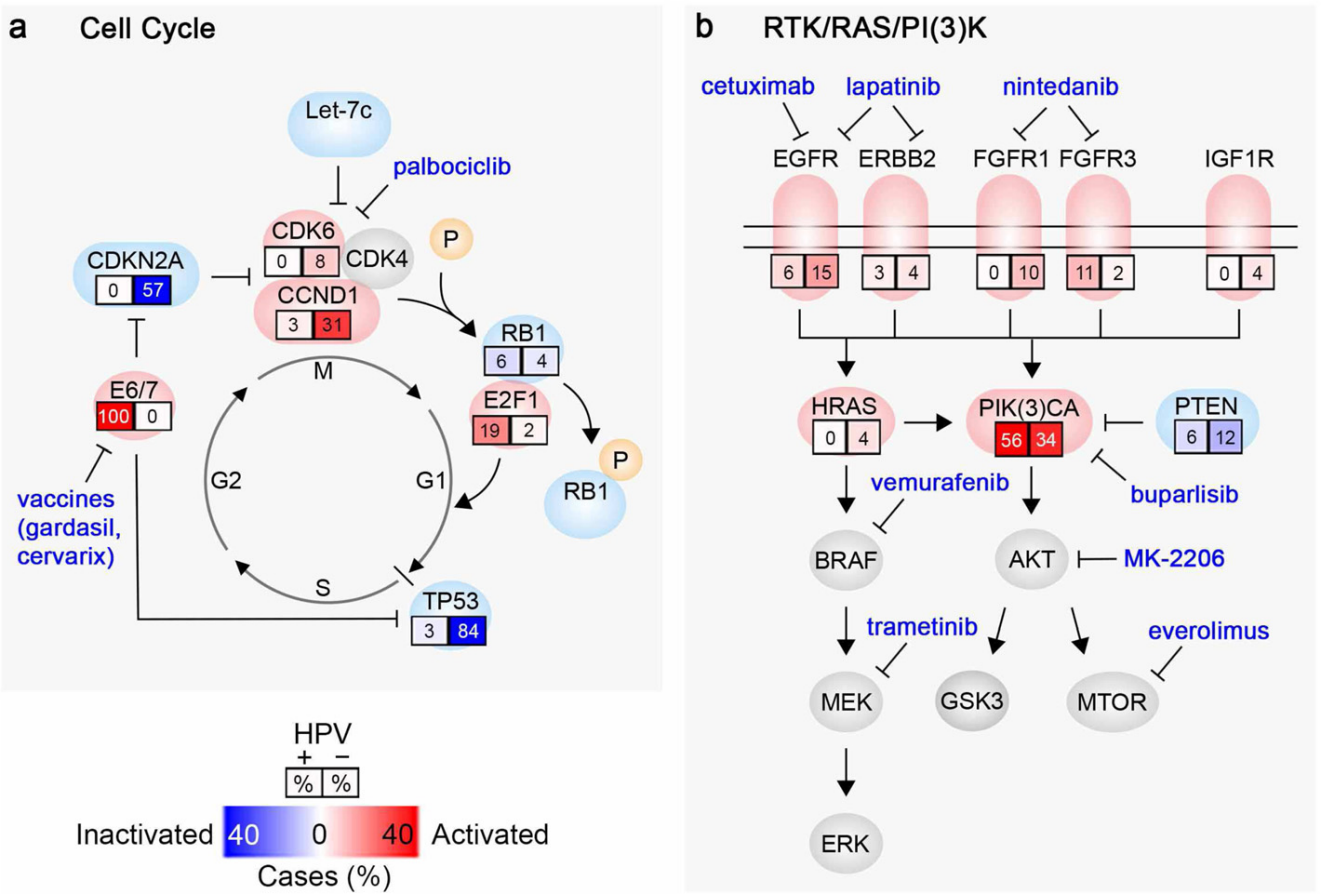
Potential therapeutic intervention based on genomic alterations. Therapeutics targeting of **a** cell cycle and **b** RTK/RAS/PI(3)K signaling associated elements. Percentages are based on the The Cancer Genome Atlas Network report [19]. RTK = receptor tyrosine kinase

The majority of patients with HNSCC are treated with surgery and/or radiation and in some cases adjuvant chemotherapy [1, 3, 11, 27]. Treatment approaches for HPV- and HPV+ cases remain very similar [1]. However, because of the better prognosis and the younger age of onset associated with HPV+ disease, therapeutic de-intensification, currently only available as part of clinical trials, for the treatment of patients with HPV+ HNSCC is being actively explored [27, 152]. Thus far, the only targeted therapeutic approved to treat HNSCC is the monoclonal antibody cetuximab, designed to target the extracellular region of EGFR (Fig. 2; [153]). The clinical impact of cetuximab has been significant in some patients [154], but relatively modest overall [2, 73, 155]. Several small molecules, for example lapatinib (targeting EGFR and HER2; [156]), afatinib (targeting EGFR and HER2; [157]) and others (reviewed in [153]), have shown some promise in the treatment of HNSCC. Inter- and intra-disease heterogeneity are likely determining factors that have thus far held back greater success of available therapeutics, and represents one of the key challenges to overcome [19, 24, 151]. Consideration of a single gene, based on a single biopsy, does not seem sufficient to maximize therapeutic interventions [73]. For example, consideration of EGFR expression and/or amplification does not correspond with response to EGFR inhibitors [107]. Data provided by the TCGA and others suggest that targeting EGFR may not be efficacious in the context of extensively altered parallel or downstream signaling components, including cell cycle regulators, due to overlapping functional contributions [73, 158].

Figure 2 summarizes potentially promising targets other than EGFR, based on available genomics data. The drugs shown in Fig. 2 are examples of drugs currently in clinical development for the treatment of HNSCC; recent reviews provide more complete lists of available drugs for each target [3, 159–161]. The near universality of cell cycle dysregulation in HNSCC strongly recommends investigation of CDK inhibitors [19, 56]. HPV- HNSCC with functional CDKN2A and high levels of phosphorylated RB1 may present the ideal molecular background for effective treatment with CDK4/6 inhibitors (Fig. 2; [56]). Furthermore, therapeutic targeting of aberrant cell cycle activity may partially circumvent the challenge presented by heterogeneity, given that clonal status analysis of the TCGA HNSCC cohort indicated that genes associated with cyclin-dependent kinases have 0 % of mutations arise in subclonal populations [24], which suggests that cell cycle alterations arise early during tumor development and are present in most if not all tumor cells. A large number of CDK inhibitors are currently in development [162] and the possibility of RB1 phosphorylation status as a response predictive biomarker is encouraging [56]. PI3K [NCT01816984], FGFR [NCT02558387], BRAF [NCT01286753], MEK [NCT01553851], AKT [NCT01349933] and mTOR [NCT01051791] are further targets of potential therapeutic relevance (Fig. 2b; [102, 103, 163, 164]). Additional promising pre-clinical work has explored Second Mitochondria-derived Activator of Caspases (SMAC)-mimetics, antagonists of inhibitors of apoptosis, which seem particularly effective against HNSCC models with FADD/BIRC2 alterations [165, 166]; particularly meaningful considering the aforementioned high incident of FADD/BIRC2 alterations in HNSCC (Table 1).

The perhaps most exciting recent development in the treatment of cancer is immunotherapy [167]. Immunotherapy, specifically checkpoint blockade, has been tremendously successful in some cases of non-small cell lung cancer [168, 169], malignant melanoma [170] and other cancers [171, 172]. Checkpoint inhibitors seem to be particularly effective against tumors with high rates of mutation, which suggests that a subpopulation of patients with HNSCC would benefit form this type of therapy. Furthermore, HNSCC appears to be an immunosuppressive disease commonly associated with lymphopenia [173, 174] and in a few cases (7 % of HPV- and 11 % of HPV+ HNSCC in the TCGA cohort) presenting with specific mutations in HLA alleles and the antigen processing machinery to reduce tumor immunodetection [19]. A substantial number of clinical trials are currently exploring the applicability of immunotherapy for the treatment of HNSCC, with primary focus on immune checkpoint blockade via CTLA-4 and PD1 [12]. In brief, CTLA-4 and PD1 are expressed by T-cells and function as negative regulators of T-cell activity, a process required for normal immunologic homeostasis. Tumor cells frequently engage CTLA-4 or PD1 to modulate T-cell activity and escape immunodetection [172, 175]. Immune checkpoint blockade inhibits interaction of tumor cells with CTLA-4 or PD1; thus, blocking inactivation of T-cells [175]. Regarding HNSCC, several phase III studies are currently exploring the utility of checkpoint inhibitors; specifically, the humanized monoclonal PD-1 specific antibody pembrolizumab [NCT02564263, NCT02358031, NCT02252042], recently approved for the treatment of melanoma and lung cancer (two cancer types with high mutational burden; [25, 26, 176–178]) and tremelimumab (fully human antibody against CTLA-4) with or without durvalumab (Fc optimized monoclonal antibody against the PD1 ligand 1; NCT02551159). Initial results are expected to be published in the near future [12]. It will be important to determine if distinct molecular lesions found in HNSCC, as summarized above, are prognostic for response to these new treatments. In the case of immunotherapy, considerations beyond the tumor may also be particularly important; for example, early laboratory studies have shown that the composition of the intestinal microbiota significantly impacts the efficacy of CTLA-4/PD-1 inhibitors [179, 180].

### Methylation in HNSCC

Future endeavors are likely to include more extensive elucidation of the role of DNA methylation in HNSCC, in part to substantiate publications based on the TCGA dataset. DNA methylation is important in the regulation of gene expression, and aberrant methylation has been described for essentially all cancer types and as a critical aspect of cancer genomics [181, 182]. Previously published work suggests that HPV+ HNSCC has significantly differentiated CpG island methylation compared to HPV- cases, reflecting the notion that HPV+ and HPV- HNSCC are distinct diseases on the genomic, transcriptomic and methylomic level [183–185]. Comparative analyses of available HPV+ TCGA cases revealed specific hypermethylated regions downstream of CDKN2A, which correlated with increased transcription of CDKN2A variant p14 (ARF; [184]). CDKN2A is also frequently methylated (23–67 % of cases; [91]) to silence expression of this tumor suppressor [88, 90, 186]; although, degree of methylation and expression changes can vary significantly among individual tumors [187]. The mechanistic and clinical ramifications of this observation are not yet understood. Another study of HPV+ HNSCC reported that a promoter methylation signature of 5 genes, three with high methylation (GATA4, GRIA4, IRX4) and two with low methylation (ALDH1A2 and OSR2), correlated strongly with improved survival [188]. The signature was validated across multiple cohorts. Methylation patters in HPV+ HNSCC are significantly distinct for cases with integrated HPV DNA and episomal DNA [34, 119], a potentially important factor not always considered.

Interestingly, prominent differential methylation of three members of the zinc finger gene family, ZNF14, ZNF160 and ZNF420, has been identified as suitable to detect HNSCC with 100 % specificity in primary tissue and saliva samples; subsequently, the three ZNF methylation signature was validated using the 273 TCGA cohort [185]. For most of the methylomics driven studies of HNSCC [183, 185, 188, 189], few cases were analyzed (particularly for HPV+ cases) and additional work is needed to better understanding and interpret the various methylation patterns. How methylomics data is going to be integrated into clinical practice for HNSCC remains to be seen, although prognostic and diagnostic potential of such information is apparent in some cancer types [181, 185, 190]. No DNA methylation markers for HNSCC have been accepted for clinical use to date [185].

## Conclusions

Detailed profiling of HNSCC by the TCGA network and other research groups has greatly enhanced our understanding of this malignancy. First and foremost, the composite results have highlighted the tremendous inter- and intra-tumor heterogeneity, complicated by the increasing incidence of HPV-associated tumors. Efforts have started to focus on classifying tumors based on molecular profiles [191–193]; however, inroads in terms of improved survival have not substantially materialized yet. The next phase is likely to require multi-platform analysis of many more HPV- and HPV+ tumors, ideally sufficient to cover each anatomic site to enable actionable conclusions. In parallel, laboratory research and clinical trials have to continue to provide data that can guide therapeutic strategies based on molecularly defined parameters and higher-order interactions. Progress continues to be made and the status quo for patients with HNSCC is likely to continue to improve over the next decade.

The greatest potential therapeutic advantage to come from the detailed parsing of HNSCC heterogeneity is advanced and eventually precise treatment with immunotherapy. For example, consistent identification of tumors with highly immunogenic alterations would significantly help guide therapeutic decision-making [194]. Immunotherapy has been remarkably successful against many types of cancer, with particularly striking successes against other carcinogen-associated cancers, such as lung cancer [169, 195] and melanoma [170, 196]. High mutation burden, common for many sub-types of HNSCC [19, 24, 25], and carcinogen-associated genomic profiles seems to correlate with higher efficacy of immunotherapy [194, 197]. Leveraging and advancing current knowledge to optimize selection of HNSCC cases for treatment with immunotherapy should be a top priority and could greatly enhance the many ongoing clinical trials [12]. The perhaps most promising approach to eradicate HPV+ HNSCC is extended use of the available vaccine, which currently appears to be successful in reducing rates of cervical cancer [198, 199] and would presumably be as successful in reducing the rate of HPV+ HNSCC.

## Abbreviations

AJUBA: protein coding gene located at 14q11.2; adaptor/scaffolding protein
AKT: also known as AKT1, v-akt murine thymoma viral oncogenes homolg 1; codes for serine-threonine protein kinase and is located at 14q32.32
ALDH1A2: aldehyde dehydrogenase 1 family member A2; enzyme that catalyzes the synthesis of retinoic acid from retinaldehyde
APOBEC: apolipoprotein B mRNA editing enzyme, catalytic polypeptide-like; family of C-to-U editing cydidine deaminase enzymes located on chromosome 22
AURKA: aurora kinase A; protein is involved in cell cycle-regulation and microtubule formation; located at 20q13
BEZ-235: ATP-competitive inhibitor; imidazoquinoline derivative targeting PI3K/mTor; also known as dactolisib
BIRC2: baculoviral IAP repeat containing 2; member of a family of proteins involved in inhibiting cell death; located at 11q22
BRAF: B-Raf proto-oncogene; member of the raf/mil family of serine/theonine kinases; located at 7q34
C > T transition: a point mutation that changes the pyrimidine thymine to the pyrimidine cytosine
CASP8: caspase 8, apoptosis-related cysteine peptidase; involved in programmed cell death; located at 2q33-q34
CCF: cancer cell fraction; used to infer the size of a subpopulation affected by somatic mutations from genomic data
CCND1: cyclin D1; member of the cyclin family associated with the regulation of CDKs; located at 11q13
CDK4: cyclin-dependent kinase 4; catalytic subunit of the protein kinase complex required for G1 cell cycle progression; located at 12q14
CDK6: cyclin-dependent kinase 6; catalytic subunit of the protein kinase complex required for G1 cell cycle progression; located at 7q21–q22
CDKN2A: cyclin-dependent kinase inhibitor 2A; codes for p16; inhibitor of CDK4; at least three alternatively spliced protein coding variants have been reported; located at 9p21
chromosome p: *petit;* shorter arm of the chromosome
chromosome q: longer arm of the chromosome
CNA: copy number alteration; gains or losses of large segments of the genome
CpG: cytosine and guanine separated by exactly one phosphate; amiable to methylation
CSMD1: CUB and Sushi multiple domains 1; located at 8p23.2
CTLA4: cytotoxic T-lymphocyte-associated protein 4; immunoglobulin; transmits inhibitory signal to T-cells; located at 2q33
CTNNB1: catenin beta 1; part of adherens junctions and signaling; located at 3p21
CUL3: cullin 3; component of an E3 ubiquitin ligase complex; located at 2q36.2
DNA: deoxyribonucleic acid
E2F1: E2F transcription factor 1; involved in cell cycle control; located at 20q11.2
E6: transforming protein; targeted p53; associated with human papillomavirus infection
E7: transforming protein; targets RB1; associated with human papillomavirus infection
EAp53: Evolutionary Action score of TP53-coding variants; computational approach to estimate the risk of various TP53 mutations in order to predict survival and treatment response
EGFR: epidermal growth factor receptor; member of the ERBB family of receptor tyrosine kinases; located at 7p12
EML4-ALK: fusion gene composed of echinoderm microtubule-associated protein-like 4 and anaplastic lymphoma kinase; chromosome 2p inversion
ERBB2: erb-b2 receptor tyrosine kinase 2; also known as HER2 or NEU; no lignd-binding domain; located 17q12
ERBB3: erb-b2 receptor tyrosine kinase 3; also known as HER3; no active kinase domain; located at 12q13
ERBB4: erb-b2 receptor tyrosine kinase 4; also known as HER4; located at 2q33.3-q34
ERK: officially known as MAPK1; mitogen-activated protein kinase 1; member of the MAP kinase family; located at 22q11.21
FADD: Fas associated via death domain; adaptor protein that interacts with surface receptors to mediate apoptosis; located at 11q13.3
FAT1: FAT atypical cadherin 1; member of the cadherin superfamily; located at 4q35
FGFR1: fibroblast growth factor receptor 1; receptor tyrosine kinase; located at 8p11.23-p11.22
FGFR3: fibroblast growth factor receptor 3; receptor tyrosine kinase; located at 4p16.3
FHIT: fragile histidine triad; codes for triphospate hydrolase required for purine metabolism; located at 3q14.2
G1: gap 1 phase; first phase of the cell cycle and part of interphase
GATA4: GATA binding protein 4; member of the GATA family of zinc-finger transcription factors; located at 8p23.1-p22
GEO: Gene Expression Omnibus; public genomic data repository
GISTIC: Genomic Identification of Significant Targets in Cancer; algorithm designed to identify likely driver somatic copy-number alterations by evaluating the amplitude and frequency of events
GRIA4: glutamate receptor, ionotropic, AMPA 4; AMPA (alpha-amino-3-hydroxy-5-methyl-4-isoxazole propionate sensitive glutamate receptor; subject to RNA editing; located at 11q22
GTPase: family of enzymes that hydrolyze guanosine triphosphate (GTP); important for signal transduction in cancer cells
HLA: human leukocyte antigen; encode proteins essential to the immune system; chromosome 6
HNSCC: head and neck squamous cell carcinoma
HPV: human papillomavirus; DNA virus; HPV subtype 16 is most commonly associated with HNSCC
HPV-16: high-risk human papillomavirus subtype and the subtype most commonly associated with cervical, anal and oropharyngeal cancer
HRAS: Harvey rat sarcoma viral oncogenes homolog; member of the Ras oncogenes family with intrinsic GTPase activity; located at 11p15.5
IGF1R: insulin like growth factor 1 receptor; tyrosine kinase receptor; located at 11q26.3
IHC: immunohistochemistry; antibody-based tissue staining
IRX4: Iroquois homeobox 4; located at 5p15.3
*ISH*: *in situ* hybridization; uses labeled complementary DNA or RNA to localize specific DNA or RNA in tissue
KEAP1: kelch like ECH associated protein 1; interacts with NFE2L2 in a redox-sensitive manner; located at 19p13.2
let-7c: MIRLET7C also known as microRNA let-7c; short non-coding RNA involved in post-translational regulation of genes; located at 21q21.1
MAF: mutant-allele fraction; fractional of total sequenced DNA with a given mutant allele based on whole-exome sequencing
MATH: mutant-allele tumor heterogeneity; width of the mutant-allele fraction distribution normalized to the median MAF value
MEK: mitogen-activated protein kinase kinase; group of kinases also known as MAP2K; MAPKK or MKK
miRNA: micro RNA; small non-coding RNA molecule
mRNA: messenger ribonucleic acid
mTOR: mechanistic target of rapamycin; serine/threonine phosphatidylinositol kinase-related kinase; located at 1p36.2
MYC: v-myc avian myelocytomatosis viral oncogenes homolog; nuclear phosphoprotein and transcription factor; located at 8q24.21
MYH9: Nonmuscle Myosin Heavy Chain II-A (also known as NMHC-II-A); cytoskeleton protein
N classification: part of the TNM classification of malignant tumours staging notation system; describes cancer positive regional lymph nodes; ranges from N0 (tumor cells absent from regional lymph nodes) to N3 (tumor cells detected in distant lymph nodes)
NFE2L2: transcription factor; regulates expression of genes with antioxidant response elements; located at 2q31
NFkB: NFKB1; nuclear factor of kappa light polypeptide gene enhancer in B-cells 1; transcription regulator; located at 4q24
NOTCH: highly conserved signaling pathway; may also refer to NOTCH1; a type 1 single pass transmembrane receptor; located at 9q34.3
NPC: nasopharyngeal cancer; originates in the upper part of the throat behind the nose and near the base of the skull
OSCC: oral squamous cell carcinoma
OSR2: odd-skipped related transcription factor 2; located at 8q22.2
p14(ARF): alternative reading frame protein product of the CDKN2A locus; inhibits the E3 ubiquitin protein ligase MDM2 (p53 degradation)
PCR: polymerase chain reaction
PD-1: PDCD1; programmed cell death 1; member of the immunoglobulin superfamily expressed on pro-B and T cells; located at 2q37.3
PI(3)K: phosphatidylinositol-4,5-bisphosphate 3-kinase; family of intracellular enzymes
PI3KCA: phosphatidylinositol-4,5-bisphosphate 3-kinase catalytic subunit alpha; codes for p110α; the catalytic subunit; located at 3q26.3
PLK1: polo-like kinase 1; serine/threonine kinase; highly expressed during mitosis; located at 16p12.2
RB1: retinoblastoma 1; negative regulator of the cell cycle; located at 13q14.2
RNA-Seq: RNA sequencing; next-generation sequencing to detect and quantify RNA
RPPA: reverse phase protein array; high-throughput antibody-based protein detection technique
RTK: receptor tyrosine kinases; large family of transmembrane tyrosine kinases
S: synthesis phase; phase of the cell cycle during which DNA is replicated; follows G1 and precedes G2
SOX2: SRY-box 2; transcription factor; located at 3q26.3-q27
SQCC: squamous cell carcinomas
TACC3: transforming, acidic coiled-coil containing protein 3; motor spindle protein; located at 4p16.3
TCGA: the Cancer Genome Atlas
TERT: telomerase reverse transcriptase; ribonucleoprotein polymerase; maintains telomere ends; located at 5p15.33
TP53: tumor protein p53; transcription factor and tumor suppressor; located at 17p13.1
TP63: tumor protein p63; p53 family transcription factor; located at 3q28
TP73: tumor protein p73; member of the p53 family of transcription factors; located at 1p36.3
TRAF3: TNF receptor associated factor 3; member of the TNF receptor associated factor protein family; located at 14q32.32
WES: whole exome sequencing; technique for sequencing protein-coding genes
YAP1: Yes associated protein 1; nuclear effector of the Hippo pathway; located at 11q13
ZNF14: zinc finger protein 14; located at 19p13.11
ZNF160: zinc finger protein 160; located at 19q13.42
ZNF420: zinc finger protein 420; located at 19q13.12
ΔNp63α: isoform of the p53 homologue TP63 that lacks (ΔN) the transactivation domain
